# ﻿Diversification deep in the heart of Texas: seven new grasshopper species and establishment of the *Melanoplusdiscolor* species group (Orthoptera, Acrididae, Melanoplinae)

**DOI:** 10.3897/zookeys.1165.104047

**Published:** 2023-06-01

**Authors:** JoVonn G. Hill

**Affiliations:** 1 Mississippi Entomological Museum, Department of Molecular Biology, Biochemistry, Entomology, and Plant Pathology, Mississippi State University, Mississippi, USA Mississippi State University Mississippi United States of America

**Keywords:** Balcones Escarpment, biodiversity hotspot, Edwards Plateau, Texas Hill Country

## Abstract

*Melanoplusdiscolor* and *Melanopluskendalli* were previously placed in the *texanus* species group. Here seven new species are described from central Texas and the combined nine species placed into the *discolor* group based on emergence time and shape of the male terminalia and genital structures. Six of these new species are from the Edwards Plateau, a known area of high endemism. Species of the *discolor* group are inhabitants of shortgrass or mixed-grass prairies, Ashe juniper or oak savannas.

## ﻿Introduction

From 2018 to 2022, I had the good fortune to conduct surveys for grasshoppers and other insects in central Texas with my colleagues, students, and family. We explored a large portion of the Edwards Plateau, an extensive tableland in central and west central Texas that covers approximately 7,907,105 hectares and represents the southernmost portion of the Great Plains physiographic province of North America (Riskind and Diamond 1988; Toomey et al. 1999; Poole et al. 2007). Across these five summers most of our efforts were focused along the eastern and southeastern edge of the Plateau, known as the Texas Hill Country or Balcones Canyonlands, where the flat terrain of the western plateau is replaced by highly dissected steep slopes and sheer cliffs bordered by the Balcones Fault Zone.

The Edwards Plateau is a well-known hotspot of North American biodiversity with more than 100 of the 400 Texas endemics being found there (Chapman and Bolen 2020). More than 2,500 plant species have been documented there, almost 60 of which are endemic and 12 more are near endemics (Carr 2005). Uplands are vegetated in a mixture of juniper-oak savannas, mixed-grass prairies, and rock outcrop communities, whereas canyons are dominated by hardwood forests (Poole et al. 2007).

The great genus *Melanoplus* Stål (Orthoptera: Acrididae), with more than 356 species and 31 established species groups, is the largest genus of North American grasshoppers (Cigliano et al. 2023). *Melanoplus* spans a range of habitats and geographic distributions, with narrow range endemics restricted to the Florida scrub or the Rocky Mountain sky islands above 4,000 m (e.g., Knowles 2000; Woller 2017), and broadly distributed grassland generalists are found across the continent. There are approximately 200 grasshopper (Acrididae) species in 61 genera known from Texas, and approximately 60 species (more than a quarter) belong to *Melanoplus*, making it the most speciose genus in the state (Stidham and Stidham 2001, 2021; Hill 2015; Otte 2019). Some of these *Melanoplus* are placed into one of the nine species groups known from Texas, but the relationships of many of the species are poorly understood and will require phylogenetic study before placement into a group. This astounding diversity, broad ecological and distributional variations, as well as inherent differences in dispersal capabilities linked to flying and flightless species, make *Melanoplus* an interesting group for study, and a primary focus of my ongoing taxonomic research.

While identifying specimens from the first year of sampling, I discovered variation in the male genitalia of *Melanoplusdiscolor* (Scudder, 1878) and *Melanopluskendalli* (Otte, 2012), that suggested several undescribed species were represented. Characters of the male genitalia have long been used for species delineations of Melanoplinae and have been further supported by molecular evidence. (Hubbell 1932; Otte 2012; Hill 2015; Woller 2017; Huang et al. 2020). Through the subsequent years, these species became an emphasis during our expeditions. Otte (2012) described *M.kendalli* and placed it and *M.discolor* in the *texanus* species group. These two species inhabit short mixed-grass prairies and savannas in the Great Plains of North America (Brust et al. 2008; Otte 2012; pers. obs.) where their ashy brown (cinereous) dorsum and paler, yellowish brown sides provide effective camouflage among the rocky soils and short vegetation. After four years of sampling in Texas and borrowing material from several collections, I noted morphological and phenological differences that suggested that *M.discolor*, *M.kendalli*, and the species described here are likely more closely related to each other than they are to members of the *texanus* group. I feel sufficient data has been gathered to describe these new species and establish a new species group to contain them.

## ﻿Materials and methods

Most specimens examined in this study were collected by personnel of the Mississippi Entomological Museum (**MEM**) during the summer months of 2018–2022. Specimens were obtained by capturing them with a standard insect net. The captured individuals were placed into a jar containing potassium cyanide, for pinning, or 100% ethanol for future molecular work. Specimens have been given unique identifying numbers and entered in the Symbiota Collection of Arthropods Network (SCAN) database.

Other specimens examined were obtained on loan from the United States National Museum (**USNM**) or the University of Michigan Museum of Zoology (**UMMZ**). Specimens of *M.discolor* from Nebraska were provided by Mathew Brust (Chadron State University), and the type specimen of *Melanoplusdiscolor* was borrowed from the Academy of Natural Sciences of Drexel University **(ANSP)**. All type specimens of newly described species are deposited in the MEM.

The internal male genitalia, which are typically concealed within the terminalia, were either exposed upon pinning fresh specimens, or the specimen was relaxed by soaking in warm water and then the genital mass was either extruded or dissected and examined in a manner similar to (Gurney and Brooks 1959). Care should be taken when extruding the genitalia as the valves, especially those of *M.kendalli*, will spread laterally and give an altered appearance. Terminology for external morphology and male genitalia follows Carbonell (2007) and Eades (2000). Habitus and internal genitalia Images were produced using a Leica DFC 495 digital camera mounted on a Leica Z16 Microscope with motorized z-stepping. Image stacks were merged using Leica Application Suite v. 4.1.0 with the Montage Module. Images were edited using Adobe Photoshop CS6 software. Measurements were made with a Leica MZ 12.5 stereomicroscope with a reticule in the following ways:

- Body Length — Dorsally from the fastigium verticis to the distal end of the genicular lobe of the caudal femur in a parallel plane with the abdomen.
- Pronotum length — Dorsally, along the median carina.
- Male Cercus Length — Laterally, maximum possible measurement of the left cercus.
- Male Cercus Basal Width — Laterally, along the point of attachment from the dorsal to ventral margin.
- Male Mid Cercus Width — Laterally, at the mid-length of the left cercus.
- Male Cercus Apex Width — Laterally, along the distal end.
- Female Dorsal Ovipositor Valve — Laterally, from the base to the apex.
- Female Ventral Ovipositor Valve — Laterally, from the base to the apex.

## ﻿Results

**Figure 1.**
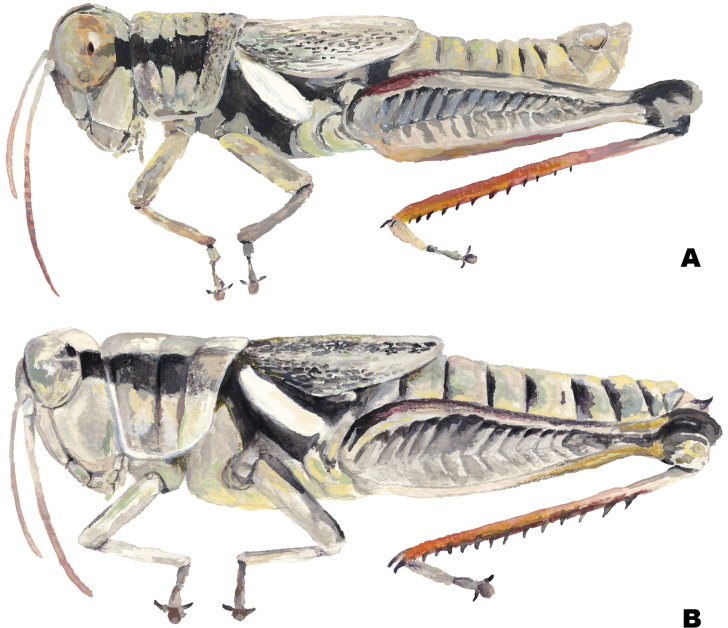
Habitus illustrations of the *Melanoplusdiscolor* group **A** male **B** female. Created by Ashley Baker.

Male specimens of *M.discolor* and *M.kendalli* collected during the 2018–2022 field seasons showed variation in the male genitalia suggesting that several undescribed species were represented. Of these species, seven species, *M.kendalli* + six undescribed species, appear to be narrow-range endemics on the Edwards Plateau. A seventh undescribed species was found on the adjacent major physiographic region, the North American Coastal Plain. These new species along with *M.discolor* and *M.kendalli* are placed in the newly formed *Melanoplusdiscolor* species group (Fig. 1) based on major differences in the shape of the male cerci, valves of the aedeagus, and time of emergence, that separates them from species in the *texanus* group.

### ﻿*Melanoplusdiscolor* group species checklist (with figure numbers presented in this work)

**Figure 2.**
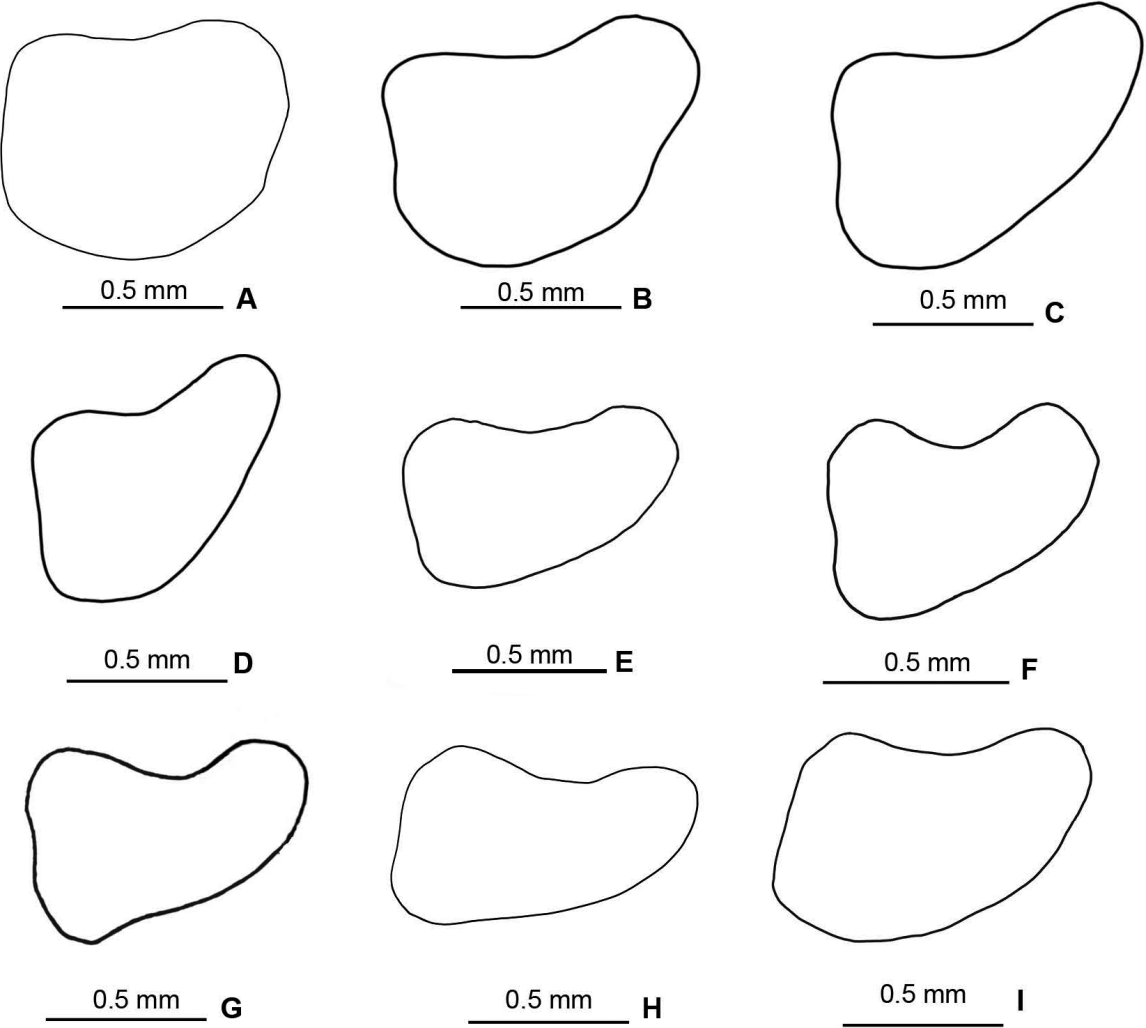
Lateral outlines of the male cerci of species in the *Melanoplusdiscolor* species group **A***M.nelsoni***B***M.discolor***C***M.corniculatus***D***M.*walkeri**E***M.walkeri***F***M.susdentatus***G***M.kendalli***H***M.tonkawa***I***M.comanche*.

**Figure 3.**
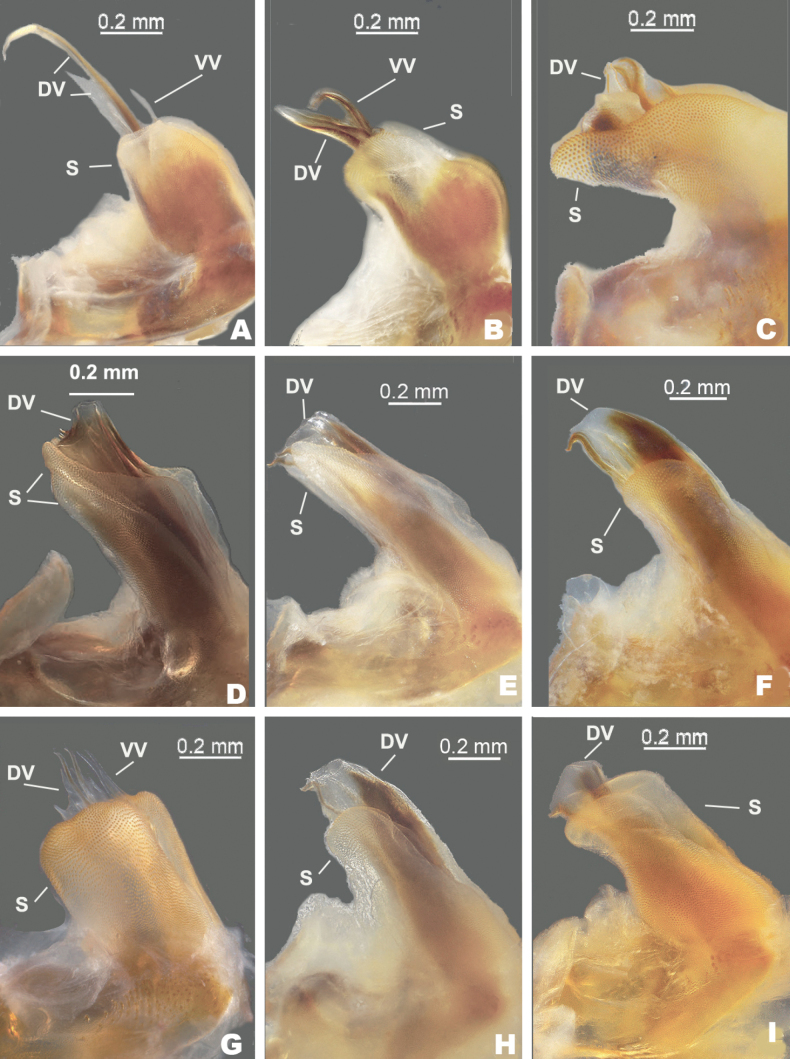
Lateral views of the male aedeagus of species in the *Melanoplusdiscolor* group. DV: dorsal valve, S: sheath, VV: ventral valve. **A***M.nelsoni***B***M.discolor***C***M.corniculatus***D***M.comanche***E***M.kendalli***F***M.susdentatus***G***M.walkeri***H***M.tonkawa***I***M.balcones*.

**Figure 4.**
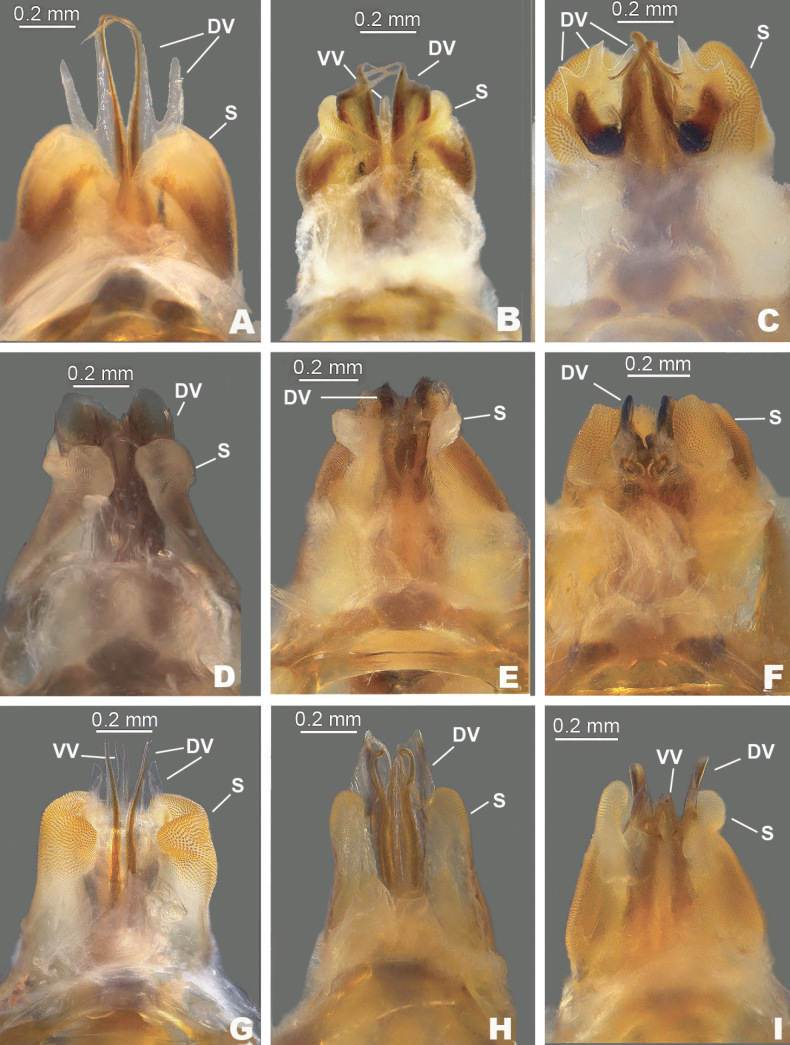
Dorsal views of the male aedeagus of species in the *Melanoplusdiscolor* group. DV: dorsal valve, S: sheath, VV: ventral valve. **A***M.nelsoni***B***M.discolor***C***M.corniculatus***D***M.comanche***E***M.kendalli***F***M.susdentatus***G***M.walkeri***H**. *M.tonkawa***I***M.balcones*.

**Figure 5.**
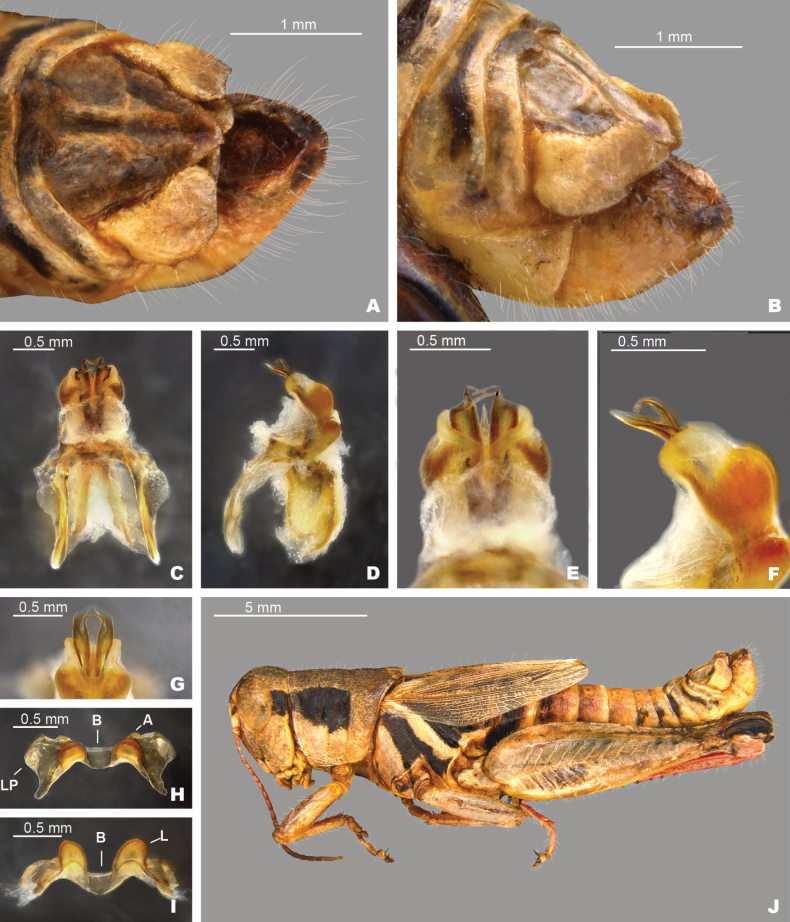
*Melanoplusdiscolor***A** dorsal view of male terminalia **B** lateral view of male terminalia **C** dorsal view of phallic complex **D** lateral view of phallic complex **E** dorsal view of aedeagus **F** lateral view of aedeagus **G** caudal view of the aedeagus **H** dorsal view of epiphallus **I** caudal view of epiphallus **J** habitus. Abbreviations: A: ancora, B: bridge, L: lophus, LP: lateral plate,

**Figure 6.**
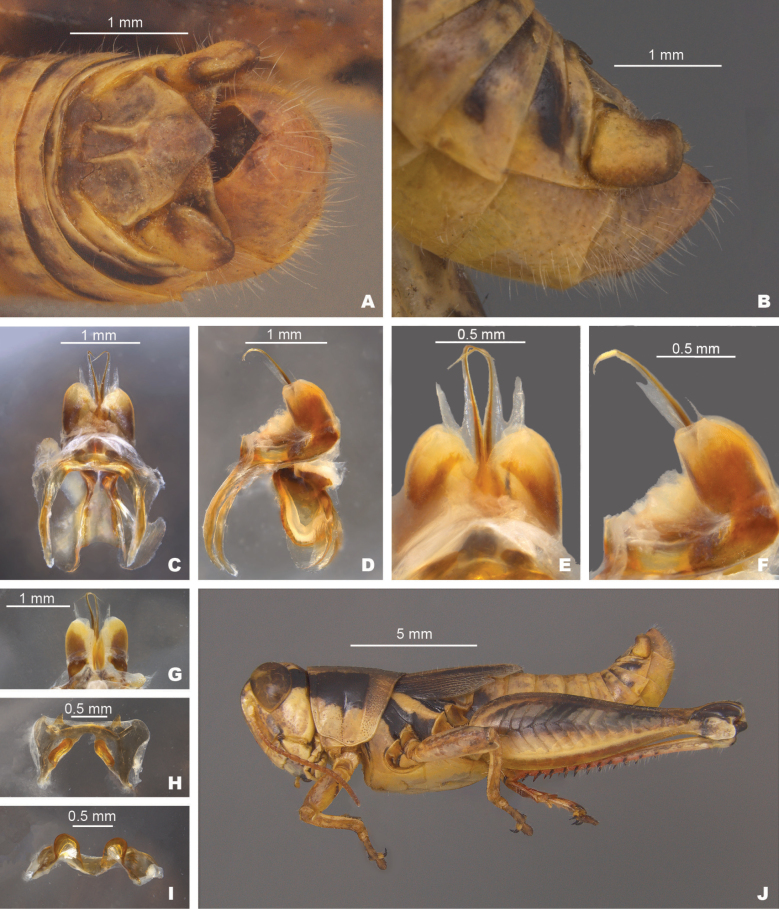
*Melanoplusnelsoni* sp. nov. **A** dorsal view of male terminalia **B** lateral view of male terminalia **C** dorsal view of phallic complex **D** lateral view of phallic complex **E** dorsal view of aedeagus **F** lateral view of aedeagus **G** caudal view of the aedeagus **H** dorsal view of epiphallus **I** caudal view of epiphallus **J** habitus.

**Figure 7.**
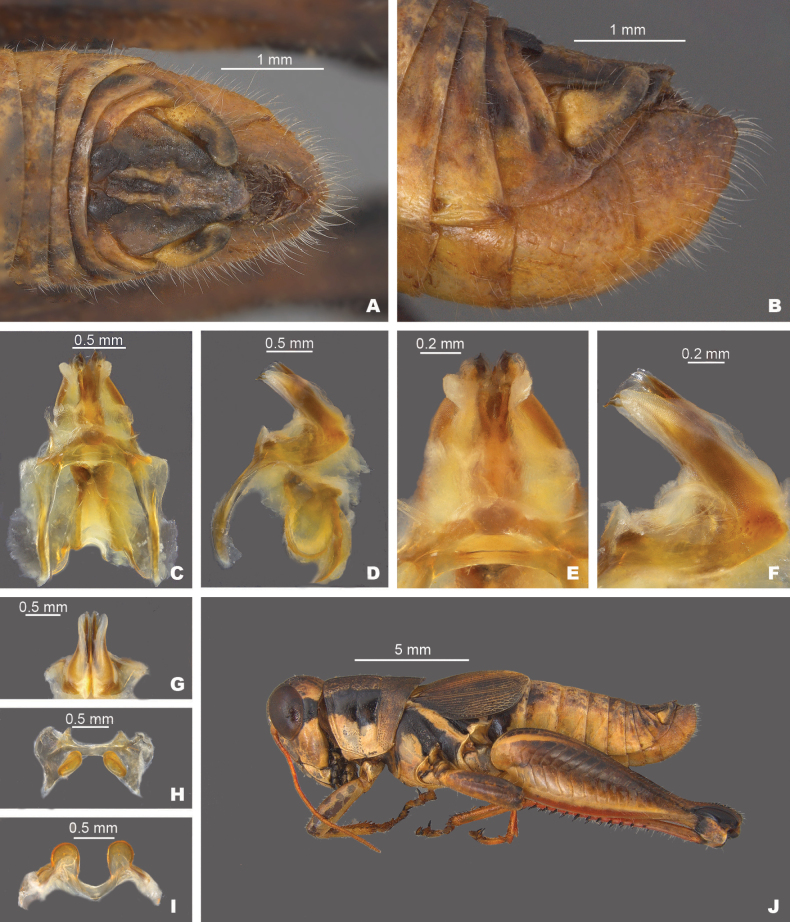
*Melanopluskendalli***A** dorsal view of male terminalia **B** lateral view of male terminalia **C** dorsal view of phallic complex **D** lateral view of phallic complex **E** dorsal view of aedeagus **F** lateral view of aedeagus **G** caudal view of the aedeagus **H** dorsal view of epiphallus **I** caudal view of epiphallus **J** habitus.

1. *Melanoplusdiscolor* (Scudder, 1878) — Figs 2B, 3B, 4B, 5A–J, 15

2. *Melanoplusnelsoni* sp. nov. — Figs 2A, 3A, 4A, 6A–H, 16

3. *Melanopluskendalli* Otte, 2012 — Figs 2H, 3F, 4F, 7A–J, 17

4. *Melanoplussusdentatus* sp. nov. — Figs 2F, 3F, 4F, 9A–J, 18

5. *Melanoplusbalcones* sp. nov. — Figs 2F, 3I, 4I, 9A–J, 19

6. *Melanopluscorniculatus* sp. nov. — Figs 2D, 3C, 4C, 10 A–J, 20

7. *Melanopluswalkeri* sp. nov. — Figs 2E, 3G, 4G, 11 A–J, 21

8. *Melanopluscomanche* sp. nov. — Figs 2G, 3D, 4D, 12A–J

9. *Melanoplustonkawa* sp. nov. — Figs 2G, 3H, 4H, 13A–J

### ﻿Comparison with related *Melanoplus* species groups occurring in Texas

#### ﻿*Melanoplusdiscolor* group

1. Hind tibia red (rarely blue);

2. Metathorax with a white stripe;

3. Pronotum below post-ocular stripe creamy yellow;

4. Tegmina lanceolate;

5. Cerci falcate with their bases much wider than their apices;

6. Dorsal valves of the aedeagus produced as variously shaped plates that occur either laterally or apical to the ventral valves;

7. Maturing late June–July and persisting until frost.

#### ﻿*Melanoplustexanus* group

1. Hind tibia red;

2. Metathorax with a white stripe;

3. Pronotum below post-ocular stripe creamy white;

4. Tegmina lanceolate;

5. Cerci variously shaped, often pyriform or subquadrate, but their bases equal to or wider than the apices;

6. Dorsal valves of the aedeagus produced to form a tubular sheath for ventral valves;

7. Maturing in April-May and persisting until July.

#### ﻿*Melanoplusscudderi* group

1. Hind tibia red;

2. Metathorax without a white stripe;

3. Pronotum below post-ocular stripe cinereous brown;

4. Cerci falcate with their bases much wider than their apices;

5. Tegmina ovate;

6. Dorsal valves of the aedeagus cylindrical and taper to a point distally;

7. Maturing in July and persisting until frost or until February in warm years.

### ﻿Key to species of the *discolor* species group

**Table:** 

1	Male cerci subquadrate to weakly falcate being about as long as wide throughout (Fig. 2A, B)	**2**
–	Male cerci falcate and often longer than wide, narrowing and curving medially to a rounded apex (Fig. 2C–I)	**3**
2	Male cerci subquadrate; aedeagus with the dorsal valves lightly sclerotized, deeply forked and the apices of the medial fork produced as a filament that is almost 2× as long as the lateral branch and curves anteriorly (Figs 3A, 4A); found in the vicinity of Bell and Burnett Counties (Fig. 14)	***nelsoni* sp. nov.**
–	Male cerci weakly falcate (Fig. 2B), dorsal valves of aedeagus more heavily sclerotized, simple, and not possessing a long filament-like structure as described above (Figs 3B, 4B); found through northern Texas north to South Dakota and west to northern New Mexico (Fig. 14)	***discolor***
3	Sheath of aedeagus projected above or equal to the dorsal height of the valves (Fig. 3C–E)	**4**
–	Sheath of aedeagus not projected past the height of the dorsal valves (Fig. 3A, B, F–I)	**6**
4	Valves of the aedeagus projected caudally; the dorsal valves are trifid with the medial branch darker and longer than the distal two (Figs 3C, 4C); sheath of the aedeagus produced to or above the dorsal margin of the valves; male cerci with a more steeply curved ventral edge as in (Fig. 2C or D); occurring in the vicinity of Kerr County (Fig. 14)	***corniculatus* sp. nov.**
–	Valves of the aedeagus projected dorsally; dorsal valves and sheath of the aedeagus variously produced; male cerci with a more broadly curving ventral edge as in Fig. 2C, F–J; found elsewhere is south-central Texas (Fig. 14)	**5**
5	Aedeagus broader in dorsal or caudal view (Fig. 4D); dorsal valves forming semi-circular like tubes (Figs 3D, 4D); cerci somewhat thicker as in Fig. 2I; found in the vicinity of Bandera County (Fig. 14)	***comanche* sp. nov.**
–	Aedeagus narrower in dorsal or caudal view (Fig. 4E); dorsal valve of the aedeagus is a sclerotized rectangular plate that is produced laterad to the ventral valves as in (Fig. 3E); male cerci with a more broadly curving ventral edge as in (Fig. 2E–I); found in vicinity of San Antonio (Fig. 14)	***kendalli***
6	Dorsal valves produced laterally to the ventral valves as heavily sclerotized plates that are angular or arching apically on their distal margin as in Figs 3E, F, H , I, 4E, F, H, I	**6**
–	Dorsal valves trifid, thinly sclerotized and projected dorsally as in Figs 3G, 4G; male cerci with a more steeply curved ventral edge as in Fig. 2C, D; found in the vicinity of Sisterdale (Fig. 14)	***walkeri* sp. nov.**
7	Caudal margin of the dorsal valves arching or swept back apically as in Fig. 3F, H	**7**
–	Caudal margin of the dorsal valves not arching apically but doing so along their distal margins as in Fig. 3I; found in the vicinity of Travis County (Fig. 14)	***balcones* sp. nov.**
8	Caudal margin of the dorsal valve longer, and broadly arching apically as in Figs 3F, 4F; Cerci slightly blunter distally (Fig. 2F); found in the vicinity of Comal and Travis Counties	***susdentatus* sp. nov.**
–	Caudal margin of the dorsal valve shorter and less sclerotized, less broadly arching apically (Figs 3G, 4G), Cerci more rounded distally (Fig. 2H); Found on the Coastal Plain in the vicinity of Fayette County (Fig. 14)	***tonkawa* sp. nov.**

### ﻿Description of species in the *Melanoplusdiscolor* group

**External morphology.** Species of medium to small size (♂ 15.5–24.5 mm, ♀ 18.5–24.5 mm). ***Head*** slightly wider than prozona; fastigum shallow and steeply declivent. Eyes somewhat prominent, especially in males. Antennae filiform, usually with 24 flagellomeres, but occasionally 25; nearly cylindrical; equal in width throughout, except two basal articles. ***Thorax*** with prosternal spine short, sub-cylindrical, broadly rounded. Pronotum broadly convex, anterior margin sub-truncate, often somewhat emarginate, the caudal margin broadly angulate. Prozona mostly smooth, with light punctation on the lobes ventrally and quadrate (more so in females) with parallel lateral margins and about 25% longer than the metazona. Metazona densely punctate, with lateral margins diverging posteriorly. Median carina distinct and equal in height throughout; anterior and median sulci present laterally, indistinct near the median carina; posterior sulci dissecting the median carina. Lateral carinae obsolete, but margins forming shoulders that range from broadly rounded on the prozona to more angular on the metazona. Lateral lobes of the pronotum quadrate with the caudal margin diverging posteriorly. Tegmina lanceolate, apices varyingly rounded; dorsal margins overlapping; dorsal and lateral fields separated by an angle; length variable but, typically extending to the anterior edge of the second abdominal tergite, some individuals with tegmina reaching the anterior edge of the third tergite. Hind femur enlarged. Metathoracic tibia with 11 or 12 pairs of spines, but typically 11. Tympanum present and obvious, appearing an opaque whitish disk. ***Terminalia of the male*** with furcula (Fig. 5A, B) typically rounded protuberances, projecting slightly beyond the end of the segment from which they originate; bases minutely separated. Supra-anal plate (Fig. 5A, B) of male triangular, length equal to the width of the base, with the median groove anteriorly distinct with elevated sides, becoming less distinct posteriorly. Cercus of the male (Figs 2, 5A, B) falciform, being broader at the base than the apex, and typically curving at the mid-point, and then tapering to rounded apex. Subgenital plate slightly conical (Fig. 5).

**Figure 8.**
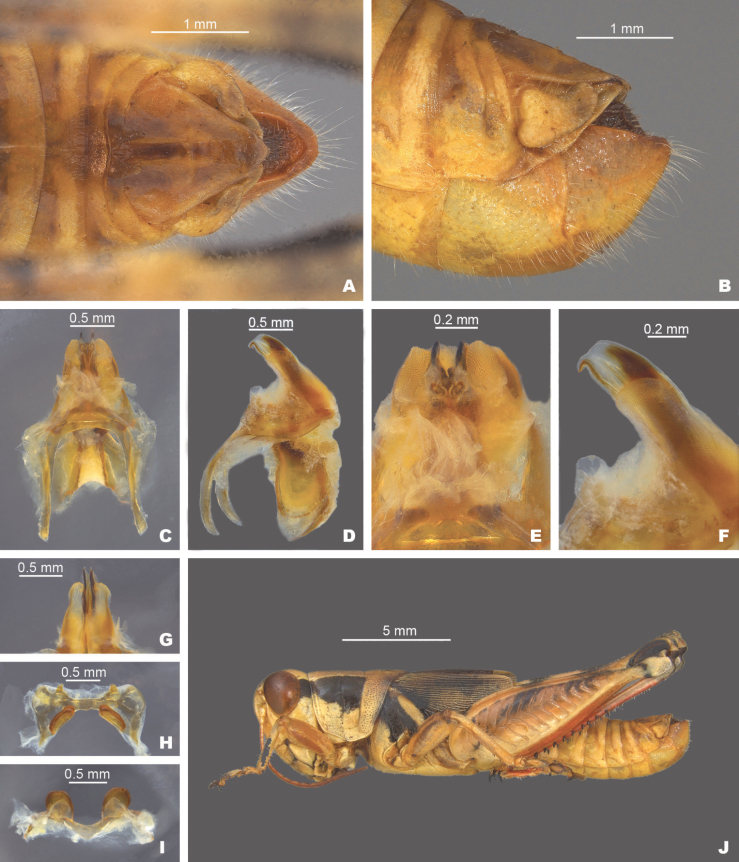
*Melanoplussusdentatus* sp. nov. **A** dorsal view of male terminalia **B** lateral view of male terminalia **C** dorsal view of phallic complex **D** lateral view of phallic complex **E** dorsal view of aedeagus **F** lateral view of aedeagus **G** caudal view of the aedeagus **H** dorsal view of epiphallus **I** caudal view of epiphallus **J** habitus.

**Figure 9.**
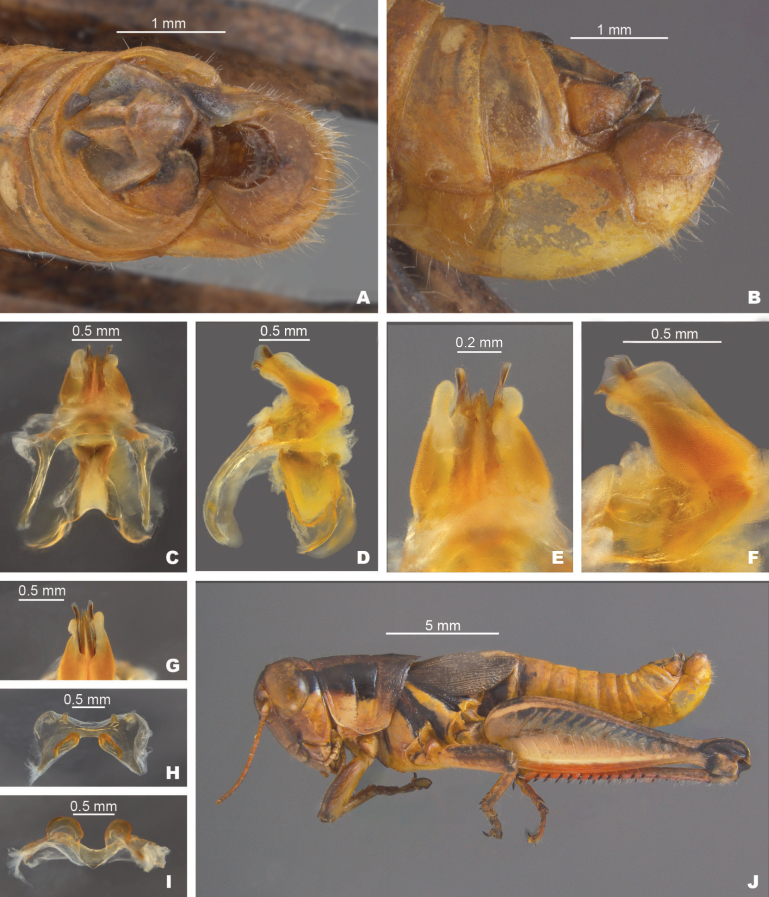
*Melanoplusbalcones* sp. nov. **A** dorsal view of male terminalia **B** lateral view of male terminalia **C** dorsal view of phallic complex **D** lateral view of phallic complex **E** dorsal view of aedeagus **F** lateral view of aedeagus **G** caudal view of the aedeagus **H** dorsal view of epiphallus **I** caudal view of epiphallus **J** habitus.

**Figure 10.**
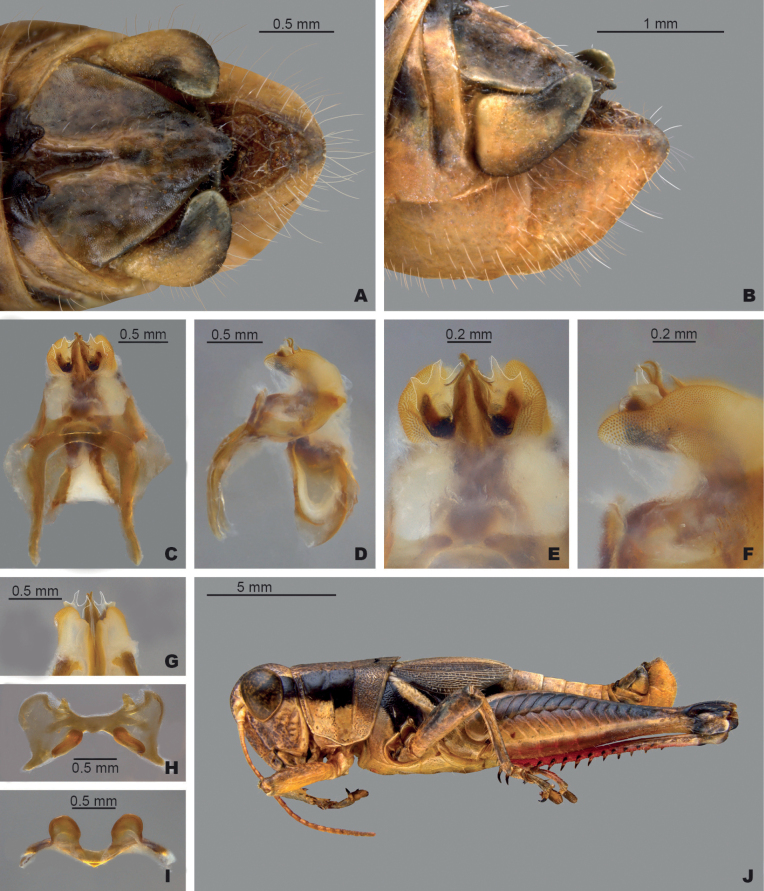
*Melanopluscorniculatus* sp. nov. **A** dorsal view of male terminalia **B** lateral view of male terminalia **C** dorsal view of phallic complex **D** lateral view of phallic complex **E** dorsal view of aedeagus **F** lateral view of aedeagus **G** caudal view of the aedeagus **H** dorsal view of epiphallus **I** caudal view of epiphallus **J** habitus.

**Figure 11.**
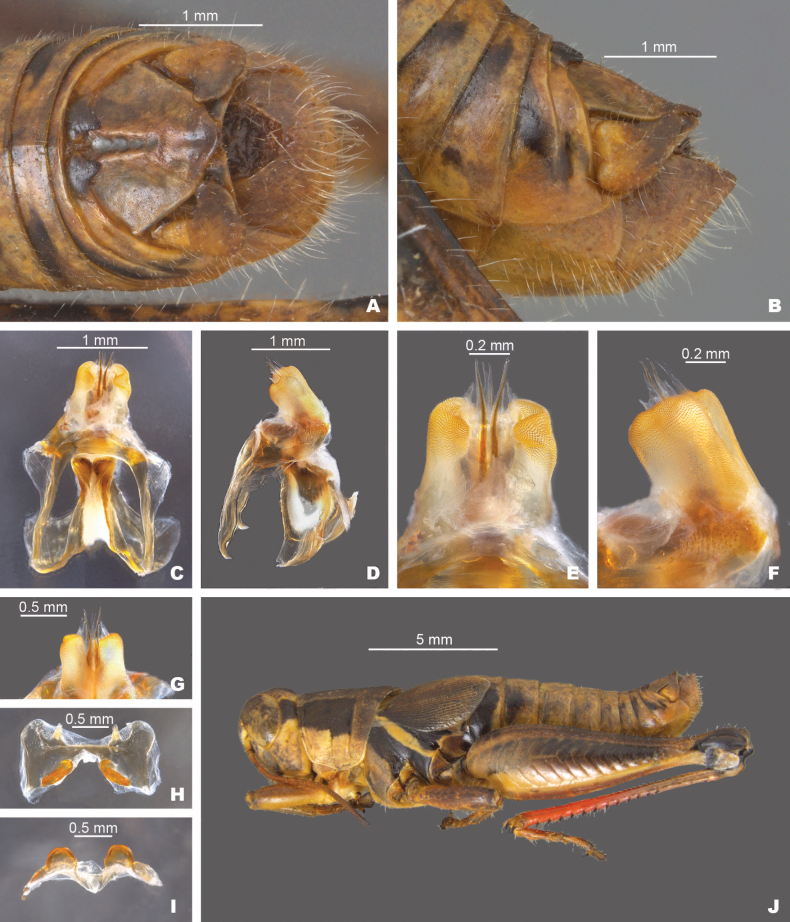
*Melanopluswalkeri* sp. nov. **A** dorsal view of male terminalia **B** lateral view of male terminalia **C** dorsal view of phallic complex **D** lateral view of phallic complex **E** dorsal view of aedeagus **F** lateral view of aedeagus **G** caudal view of the aedeagus **H** dorsal view of epiphallus **I** caudal view of epiphallus **J** habitus.

**Figure 12.**
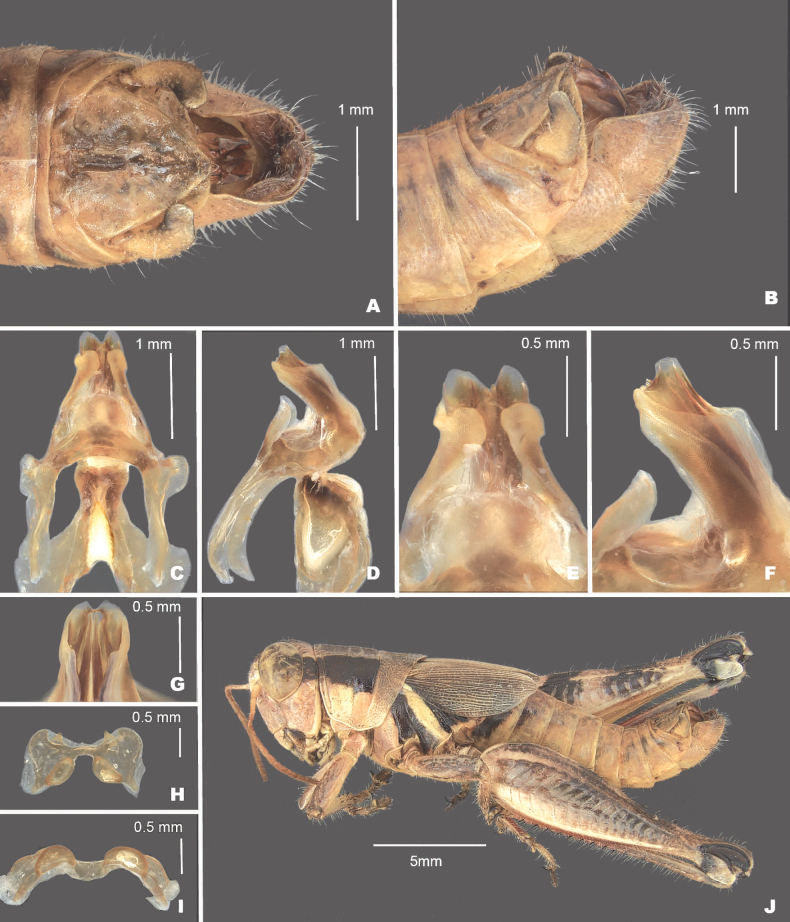
*Melanopluscomanche* sp. nov. **A** dorsal view of male terminalia **B** lateral view of male terminalia **C** dorsal view of phallic complex **D** lateral view of phallic complex **E** dorsal view of aedeagus **F** lateral view of aedeagus **G** caudal view of the aedeagus **H** dorsal view of epiphallus **I** caudal view of epiphallus **J** habitus.

**Figure 13.**
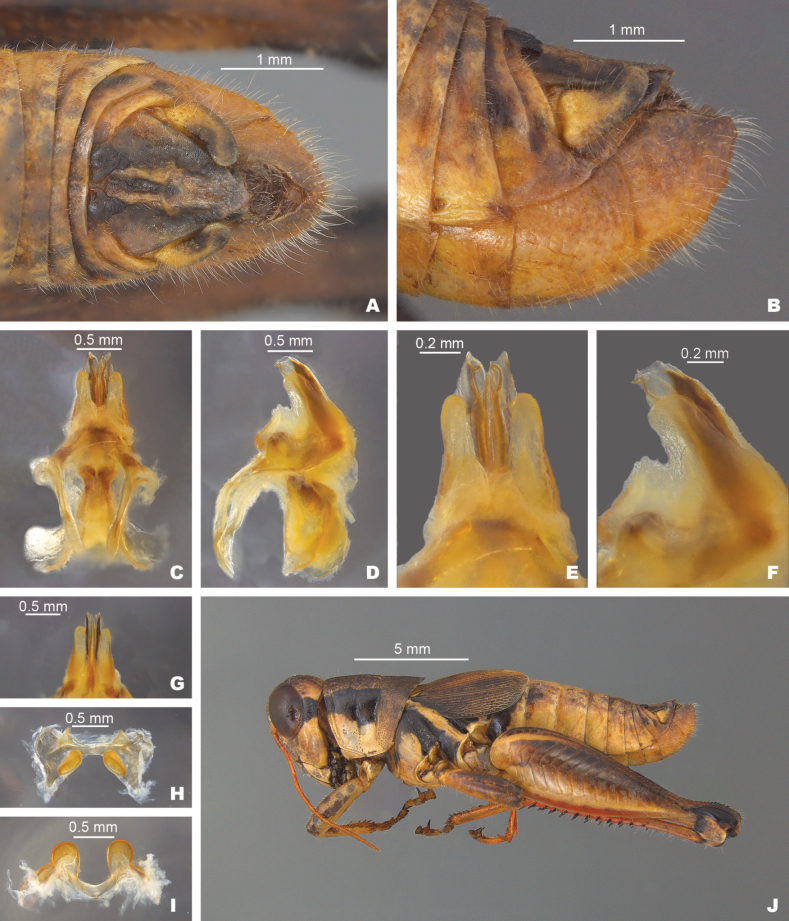
*Melanoplustonkawa* sp. nov. **A** dorsal view of male terminalia **B** lateral view of male terminalia **C** dorsal view of phallic complex **D** lateral view of phallic complex **E** dorsal view of aedeagus **F** lateral view of aedeagus **G** caudal view of the aedeagus **H** dorsal view of epiphallus **I** caudal view of epiphallus **J** habitus.

***Phallic structures*.** The dorsal valves of the aedeagus are quite variable between species, but often produced as broad plates either laterally or apical to the ventral valves. The ventral valves are cylindrical or linear processes with their distal portions curving apically (Figs 3, 4). Aedeagal sheath of various lengths depending on the species (Figs 3, 4). The epiphallus is of the typical melanoploid shape, having lophi, ancorae, and an undivided bridge, but more precisely, members of the *discolor* group have a concave bridge, broadly rounded lophi, convexly curved lateral plates that are subdeltate in shape with a rounded anterior lobe and an acuminate caudal tip, and ancora that are triangular, often tapering to a point (Figs 5–13H, I). See Fig. 5 for labeled image.

**Females**: Females are similar to the males, but differ in being larger, more robust, with broader tegmina, and in the shape of the terminalia (Figs 15C, E, 16D, 17E, F, 18G, H, 19D, 21C). ***Terminalia of female*** with triangular cerci and ovipositor valves that are subequal in length. The dorsal valves with their dorsum being nodose to slightly serrate proximally and concave and upcurving to a tip distally,

**Coloration.** Overall a yellowish or cinereous brown dorsally, a paler brownish yellow below. Antennae varying from pale red, infuscated apically to ferruginous. A lateral, well-defined, piceous, post-ocular stripe extends from the caudal margin of the eye to the second abdominal tergite, but often enfeebled or lacking on the metazona; lateral area of head, thorax, and abdomen below post-ocular stripe varying from yellowish brown to a light creamy yellow. The ridge of the metathoracic episterna marked with a light creamy white dash that is margined on either side by the black post-ocular stripe. Tegmina cinereous brown with slightly lighter venation and black infuscations, but in some individuals the dorsal fields are pale brownish yellow. Hind femur bimaculate or often feebly trimaculate dorsally, and with the lateral field infuscated with a cinereous brown band dorsally with yellowish brown below. Hind tibia red (rarely blue) with black-tipped spines (Figs 5–13J).

#### ﻿ Melanoplus discolor

Taxon classificationAnimaliaOrthopteraAcrididae

(Scudder, 1878)

3AABEF2C-AA70-540B-A9D2-6F4B922E7BF1

**Figure 14.**
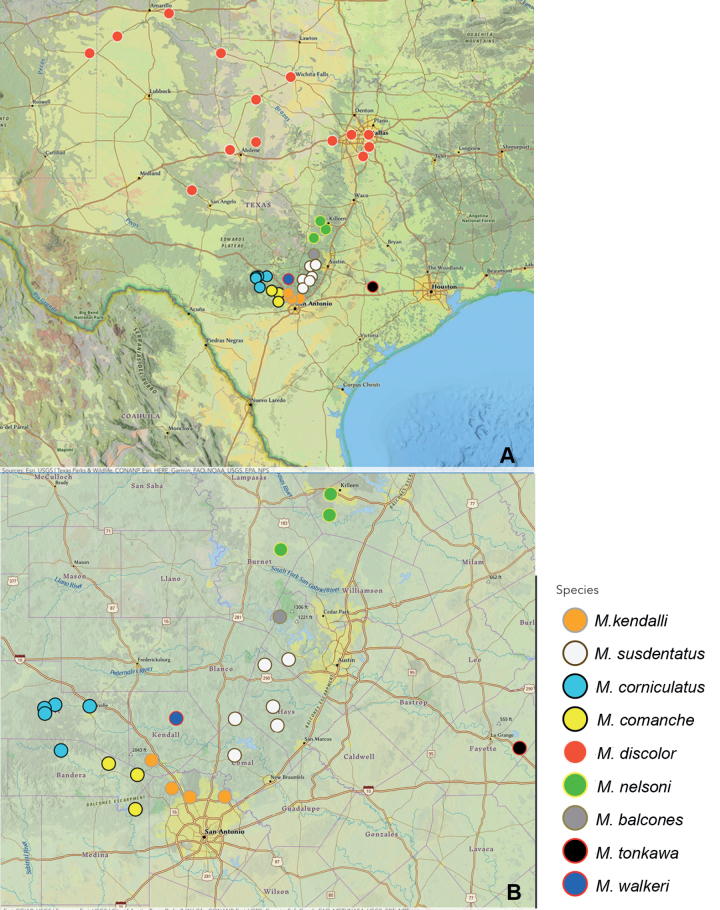
Distribution of species in the *discolor* group **A** broad view of the group, showing the wide distribution of *M.discolor***B** distribution of *discolor* group species occurring in central Texas with the Balcones Escarpment indicated.

**Figure 15.**
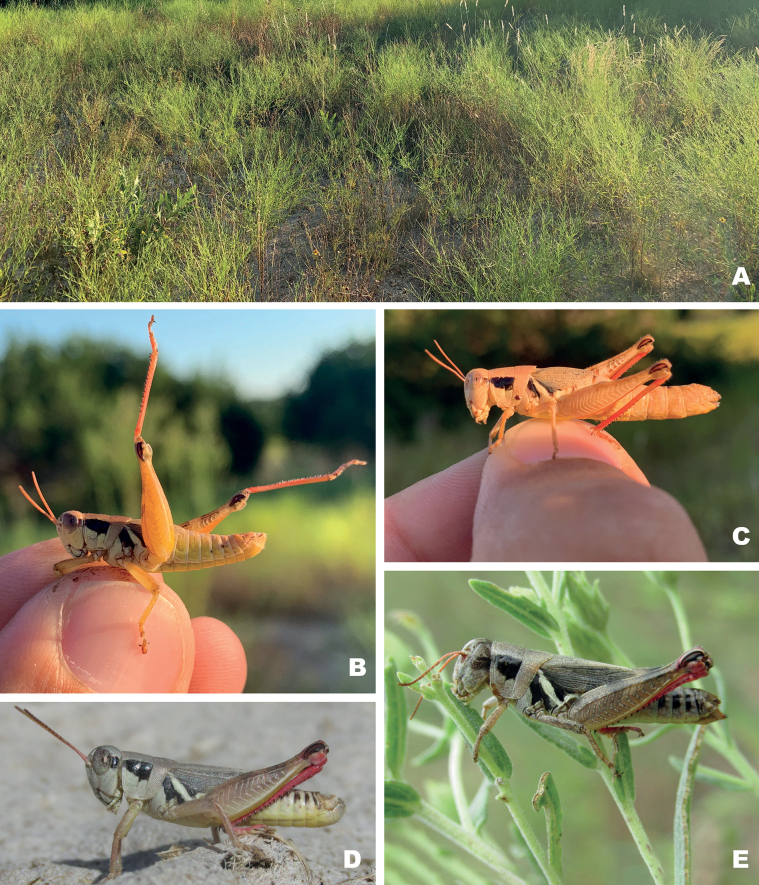
Habitat and live images of *Melanoplusdiscolor***A** prairie remnant, Ellis County, Texas **B** male, Ellis County Texas **C** female, Ellis County Texas (Note: late afternoon sun altered the body coloration slightly) **D** male, Sioux Co., Nebraska **E** female eating false boneset, Sioux Co., Nebraska. **E, D** courtesy of Matthew Brust.

Figs 2B , 3B , 4B , 5A–J , 14 , 15

Pezotettix discolor Scudder, S.H. 1878. Proc. Boston Soc. Nat. Hist. 20: 81.
Pezotettix discolor Scudder, S. H. 1897. Proc. U.S. Nation. Mus 20 (1124): 146.
Melanoplus discolor Hebard. 1917. Proc. Acad. Nat. Sci. Philad. 69: 268.
Melanoplus discolor Otte, D. 2012. Trans. Am. Ent. Soc. 138: 160.

##### Specimens examined.

**Colorado**, El Paso Co., 27 August 1938 (1♂). **Kansas**, Glasscock Co., Garden City, 23 August 1913, F.W. Milliken (1♂). Nebraska, Dawes Co, 1.7 mi SSE Whitney, off Hwy 20, 42.7001, -103.2496, 27 August 2022, M.L. Brust (4♂, 2♀), Sioux Co., ca 2.0 mi SSE Whitney of HWY 20, 42.6667, -103.5152, 26 July 2022, M.L. Brust (2♂). **New Mexico**, Curry Co., 1 September 1938 (1♂). **South Dakota**, Fall River Co., 16.5 mi N of Ardmore, Indian Canyon Rd of Hwy 71, 43.2350, -103.61975, 8 August 2020, M.L. Brust (2♂). **Texas**: Dallas Co., Dallas, C.V. Riley (1♀). Ellis Co., 3 mi SW Cedar Hill, 32.5514, -96.9911, 24 July 2019, J.G. Hill, M.J. Thorn, B.S. Dunaway (1♀). Hood Co., Cresson, 11 August 1955, Williams (1♂). Lampasas Co., 15 mi W Lampasas, 31.0417, -98.4347, 21 July 2019, J.G. Hill, M.J. Thorn, B.S. Dunaway (1♀). Shackelford Co., Albany, 20 August 1935, I.J. Cantrall (2♂). Tarrant Co., 19 July 1927 (1♂, 1♀).

##### Diagnosis.

Male cerci that are weakly falcate (Figs 2B, 5A, B), and by the shape of the internal male genitalia which has simple and heavily sclerotized dorsal valves of the aedeagus that abruptly narrow into short filamentous tails distally (Fig. 5E). The ventral valves are slightly shorter than the dorsal valves, are slightly arched posteriorly and have their distal ends bent medially (Fig. 5F). Most similar to *M.nelsoni*, but *M.discolor* is easily separated from that species by having longer and more falcate male cerci, and much shorter filamentous tails on the dorsal valves (Fig. 5C–G).

##### Measurements.

**Male measurements.** (mm): (*n* = 10) Body length 16.0–22.3 (mean = 18.8); pronotum length 3.7–5.0 (mean = 4.3); hind femur length 5.5–9.0 (mean = 7.0); cerci length 0.9–1.0 (mean = 1.0); basal width of cercus 0.5–0.8 (mean = 0.7); mid-cercal width 0.5–0.8 (mean = 0.7); cerci apex width 0.3–0.4 (mean = 0.3).

**Female measurements.** (mm): (*n* = 8) Body length 18.5–25.2 (mean = 21.3); pronotum length 4.3–6.3 (mean = 5.1); tegmen length 6.5–9.9 (mean = 8.0); hind femur length 10.5–15.8 (mean = 12.6) Dorsal ovipositor valve length 1.3–1.6 (mean = 1.5); ventral ovipositor valve length 1.0 (mean = 1.0).

##### Habitat.

Shortgrass or mixed grass prairie and Ashe juniper savannas (Fig. 15A).

##### Distribution.

North Texas through the Great Plains to South Dakota west to New Mexico and Colorado (Fig. 14).

##### Etymology.

Discolor, Latin, of different colors or variegated.

##### Suggested common name.

Variegated pouncer.

##### Notes.

Specimens north of Texas tend to have a deeper red hind tibia (Fig. 15D, E), This species has been observed eating *Brickelliaeupatorioides*, false boneset (Asteraceae) (Fig. 15E).

#### ﻿ Melanoplus nelsoni sp. nov.

Taxon classificationAnimaliaOrthopteraAcrididae

E3C50829-0E07-534E-9877-B913299842F5

https://zoobank.org/225DA1B7-5356-440C-9421-C010583D8421

**Figure 16.**
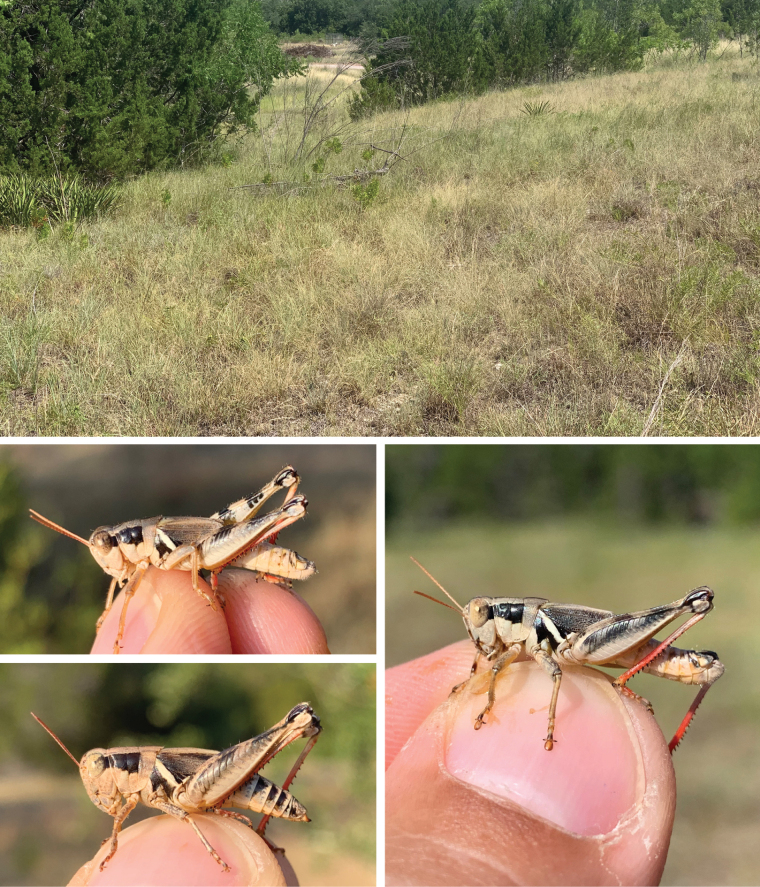
Habitat and live images of *Melanoplusnelsoni* sp. nov. **A** Ashe juniper savanna, Burnett County, Texas **B** male **C** female **D** male.

Figs 2A , 3A , 4A , 6A–H , 14 , 16

##### Type material.

***Holotype*.** 1♂, USA, TX, Bell Co., 1.7 mi N Ding Dong, 30.9969, -97.7861, 4 October 2019, J.G. Hill, MJT, BSD; Collected in open Ashe juniper woodland; MEM 281,626. Deposited in the Mississippi Entomological Museum.

##### Specimens examined.

**Texas**: Bell Co., 1.7 mi N Ding Dong, 30.9969, -97.7861, 4 October 2019, J.G. Hill and M.J. Thorn (3♂, 5♀), 3 mi W Killeen, Hood Village, 26 August 1955, T.J. Cohn (2♂). Burnet Co., 6 mi E Burnet, 9 July 1959, T.J. Cohn (3♂); 2 mi W Joppa, 30.8298, -98.0608, 13 July 2020, J.G. Hill (8♂, 2♀).

##### Diagnosis.

Easily differentiated from the other species in the group based on the male cerci which are subquadrate (Figs 2A, 6A, B) and by the male aedeagus, which has lightly sclerotized dorsal valves that are deeply forked and with the apices of the medial fork produced as a filament tail that is almost two times as long as the lateral branch and curves anteriorly (Fig. 6E). Most similar to *M.discolor*, but *M.nelsoni* is easily distinguished from that species by having shorter more quadrate male cerci and much longer filamentous tails on the dorsal valves (Fig. 6C–G).

##### Measurements.

**Male measurements.** (mm): (*n* = 12) Body length 15.5–22.5 (mean = 20.1); pronotum length 3.3–5.1 (mean = 4.3); tegmen length 4.6–7.1 (mean = 5.7); hind femur length 9.0–12.3 (mean = 11.0); cerci length 0.7–1.0 (mean = 0.9); basal width of cercus 0.5–1.0 (mean = 0.8); mid-cercal width 0.5–0.6 (mean = 0.6); cerci apex width 0.3–0.4 (mean = 0.4).

**Female measurements.** (mm): (*n* = 9) Body length 19.5–24.0 (mean = 21.6); pronotum length 4.2–5.7 (mean = 5.0); tegmen length 5.5–7.5 (mean = 6.5); hind femur length 10.5–14.0 (mean = 12.4) Dorsal ovipositor valve length 1.0–1.5 (mean = 1.3); ventral ovipositor valve length 1.0–1.5 (mean = 1.3).

##### Habitat.

Ashe juniper savannas with short vegetation, exposed rocks, and bare ground (Fig. 16A).

##### Distribution.

The northeastern Edwards Plateau in Bell and Burnet Counties, Texas (Fig. 14).

##### Etymology.

Named in honor of Willie Nelson, an iconic American musician entertainer from central Texas whose music lifted our spirits while traveling between field sites during this study. After these last few summers, just like Mr. Nelson, we too have a little Texas in our souls.

##### Suggested common name.

Nelson’s pouncer.

#### ﻿ Melanoplus kendalli

Taxon classificationAnimaliaOrthopteraAcrididae

Otte, 2012

CF557F4A-7939-547E-85A5-B2CD4F3257A1

**Figure 17.**
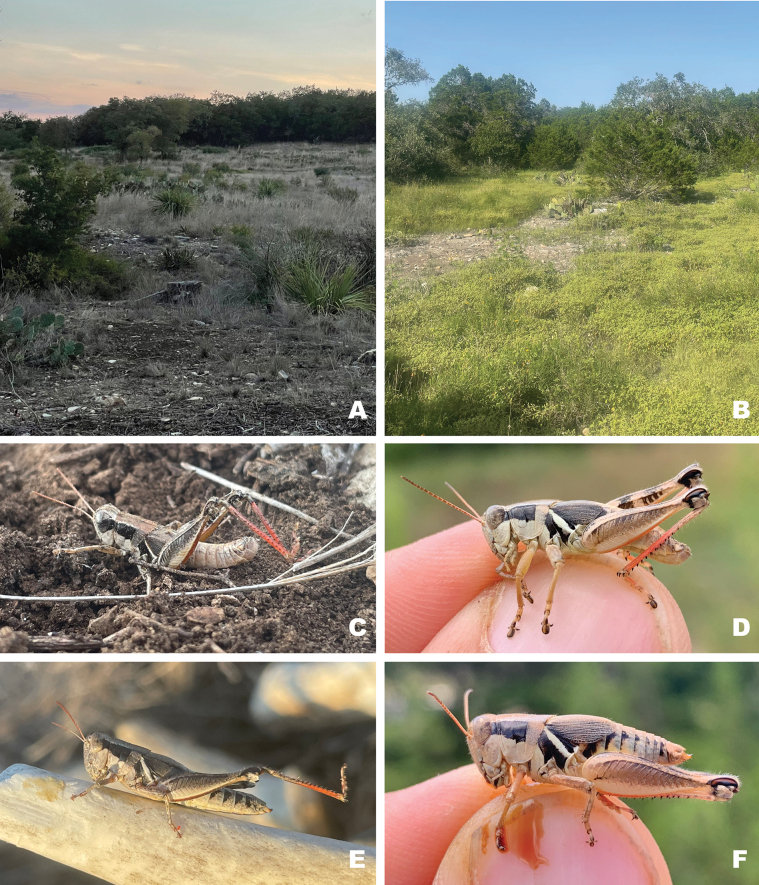
Habitat and live images of *Melanopluskendalli***A** Ashe juniper savanna, Bexar County, Texas **B** Ashe juniper savanna, Bexar, County Texas **C** male **D** male **E** female **F** female.

Figs 2H , 3F , 4F , 7A–J , 14 , 17

Melanoplus kendalli Otte, D. 2012. Trans. Am. Ent. Soc. 138:160.

##### Specimens examined.

**Texas**: Bexar Co., 2 mi W Bracken, 29.6235, -98.3774, 26 August 2022, J.G. Hill, J.R. Fisher (3♂, 3♀); Eisenhower Park, 29.6220, -98.5735, 25 August 2022, J.G. Hill, J.R. Fisher (4♂, 2♀); 2 mi N Leon Springs, 29.6636, -98.6761, 16 July 2019, J.G. Hill, M.J. Thorn, B.S. Dunaway (5♂, 4♀). Kendall Co., 2 mi NW Boerne, 20 July 1959, T.J. Cohn (4♂, 1♀). Medina Co., 3 mi NE Mico, 29.5613, -98.8792, 21 July 2020, A.G. Hendon (1♂), same data except, M.J. Thorn (2♂, 1♀).

##### Diagnosis.

Male cerci broadly falcate (Figs 2H, 7A, B), internal male genitalia with the aedeagal sheath projected to the distal edge of the apical side of the dorsal valves. Dorsal valves are thin rectangular plates that are produced laterally to the ventral valves, giving the aedeagus a narrow or thin appearance in caudal or dorsal views (Figs 3E, 4E). The ventral valves are slightly shorter than the dorsal valves, are slightly arched posteriorly and have their distal ends bent medially (Fig. 7F). Most similar to *M.susdentatus* and *M.comanche*, but *M.kendalli* is easily separated from those species by having an aedeagal sheath that reaches the distal margin of the dorsal valves and the thin, rectangular nature of the dorsal valves (Fig. 7C–G).

##### Measurements.

**Male measurements.** (mm): (*n* = 15) Body length 19.1–23.5 (mean = 20.7); pronotum length 4.1–5.2 (mean = 4.5); tegmen length 4.2–5.5 (mean = 4.9); hind femur length 10.1–12.5 (mean = 11.5); cerci length 0.8–1.1 (mean = 0.9); basal width of cercus 0.5–0.7 (mean = 0.6); mid-cercal width 0.3–0.4 (mean = 0.4); cerci apex width 0.2–0.4 (mean = 0.3).

**Female measurements.** (mm): (*n* = 10) Body length 20.1–24.5 (mean = 22.7); pronotum length 4.2–5.6 (mean = 5.0); tegmen length 4.5–6.0 (mean = 5.5); hind femur length 11.1–14.0 (mean = 12.6) Dorsal ovipositor valve length 1.2–1.6 (mean = 1.4); ventral ovipositor valve length 1.0–1.6 (mean = 1.2).

##### Habitat.

Ashe juniper savanna with short grasses (Fig. 17A, B) and post oak savanna.

##### Distribution.

Southeastern Edwards Plateau/Balcones Escarpment in the vicinity of Bexar, Kendall, and Medina Counties (Fig. 14).

##### Suggested common name.

Otte (2012) does not give the etymology of the name at the time of the description, but in recent conversation, Otte told me that it was because it was from Kendall County. Kendall County is named after George Wilkins Kendall, a journalist and war correspondent during the Mexican-American War. However, given that the epithet is *kendalli* and not *kendallensis*, I suggest Kendall’s pouncer as the common name.

#### ﻿ Melanoplus susdentatus sp. nov.

Taxon classificationAnimaliaOrthopteraAcrididae

86D77B27-A47E-5F0B-A2EF-D8B51F8F4701

https://zoobank.org/E380D24E-3C39-4E0F-AF19-2B9743B48B46

**Figure 18.**
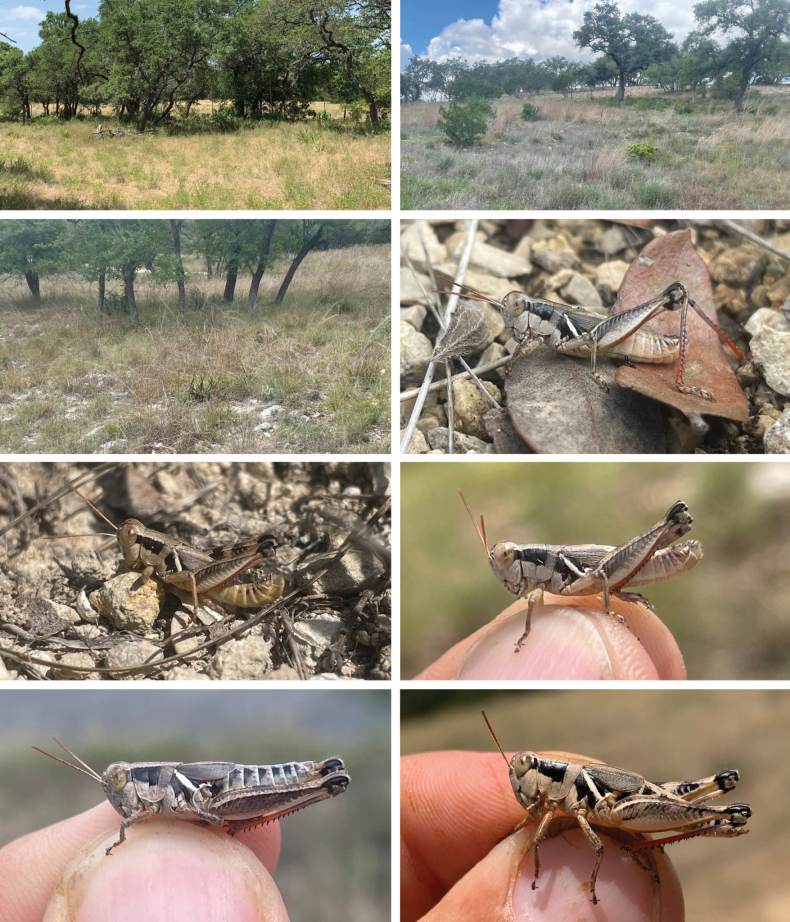
Habitat and live images of *Melanoplussusdentatus* sp. nov. **A** oak savanna, Hayes Co., Texas **B** oak savanna, Travis Co, Texas **C** oak savanna, Comal Co., Texas **D** male **E** male **F** male **G** female **H** female.

Figs 2F , 3F , 4F , 9A–J , 14 , 18

##### Type material.

***Holotype***: 1♂, USA, TX, Comal Co. 3.8 mi NW FIsher, 30.0051, -98.3190, 16 July 2020, J.G. Hill; Ashe juniper savanna. Deposited in the Mississippi Entomological Museum.

##### Other specimens examined.

Texas: Comal Co., 6.6 mi E Bulverde, 29.8283, -98.3127, 26 August 2022, J.G. Hill (3♂, 1♀); 3.8 mi Fisher, 30.0051, -98.3190, 16 July 2020, J.G. Hill (2♂). Hayes Co., 6.7 mi NE Dripping Springs, 30.269, -98.1506, 28 August 2022, J.G. Hill, J.R. Fisher (3♂, 1♀); 3.96 mi SW Pioneer Town, 29.9591, -98.1662, 2 August 2021, J.G. Hill (1♂); 2.4 mi N Woodcreek, 30.0635, -98.1063, 23 July 2021, J.G. Hill (1♂); 2 mi S Wimberly, 5 August 1953, N.W. Wasseman (1♂); 4.5 mi N Wimberly, 30.0635, -98.1061, J.G. Hill (5♂, 2♀). Travis Co., 4.12 mi W Bee Cave, 30.2945, -98.0182, 29 August 2022, J.G. Hill, J.R. Fisher (4♂, 1♀).

##### Diagnosis.

Male cerci that are broadly falcate (Figs 2F, 9A, B), and the internal male genitalia with the aedeagal sheath that does not project to the distal edge dorsal valves. Dorsal valves are thin plates that are arched along the caudal margin and are produced laterally to the ventral valves, giving the aedeagus a narrow or thin appearance in caudal or dorsal views (Figs 3F, 4F). The ventral valves are slightly shorter than the dorsal valves (Fig. 9F). Most similar to *M.kendalli* and *M.balcones*. *Melanoplussusdentatus* is easily separated from *M.kendalli* by having an aedeagal sheath that does not reach the distal margin of the dorsal valves and the curved nature of the dorsal valves (Figs 8C–G, 9C–G), and from *M.balcones* by the shorter, rounded valves found in that species (Fig. 9D, F).

##### Measurements.

**Male measurements.** (mm): (*n* = 10) Body length 17.5–21.5 (mean = 19.6); pronotum length 3.5–4.7 (mean = 4.1); tegmen length 4.1–5.1 (mean = 4.5); hind femur length 9.6–11.5 (mean = 10.6); cerci length 0.8–1.0 (mean = 1.0); basal width of cercus 0.5–0.6 (mean = 0.6); mid-cercal width 0.4–0.5 (mean = 0.5); cerci apex width 0.2–0.3 (mean = 0.3).

**Female measurements.** (mm): (*n* = 4) Body length 18.5–21.1 (mean = 19.9); pronotum length 4.2–4.7 (mean = 4.5); tegmen length 4.1–5.9 (mean = 5.0); hind femur length 10.7–12.3 (mean = 11.7) Dorsal ovipositor valve length 1.2–1.3 (mean = 1.3); ventral ovipositor valve length 1.0 (mean = 1.0).

##### Habitat.

Ashe Juniper savanna (Fig. 18A).

##### Etymology.

From the Latin *sus* for hog and *dentatus* for toothed. In reference to the dorsal valves being shaped like the distal ends of the incisors and tusk of *Susscrofa* L., a notorious invasive species in Texas.

##### Suggested common name.

Hog-toothed pouncer.

#### ﻿ Melanoplus balcones sp. nov.

Taxon classificationAnimaliaOrthopteraAcrididae

52A4AEC0-8C2B-5F73-9D5B-4DA055D1ACC9

https://zoobank.org/91E030AE-061E-4762-92C8-62ABDCB30F93

**Figure 19.**
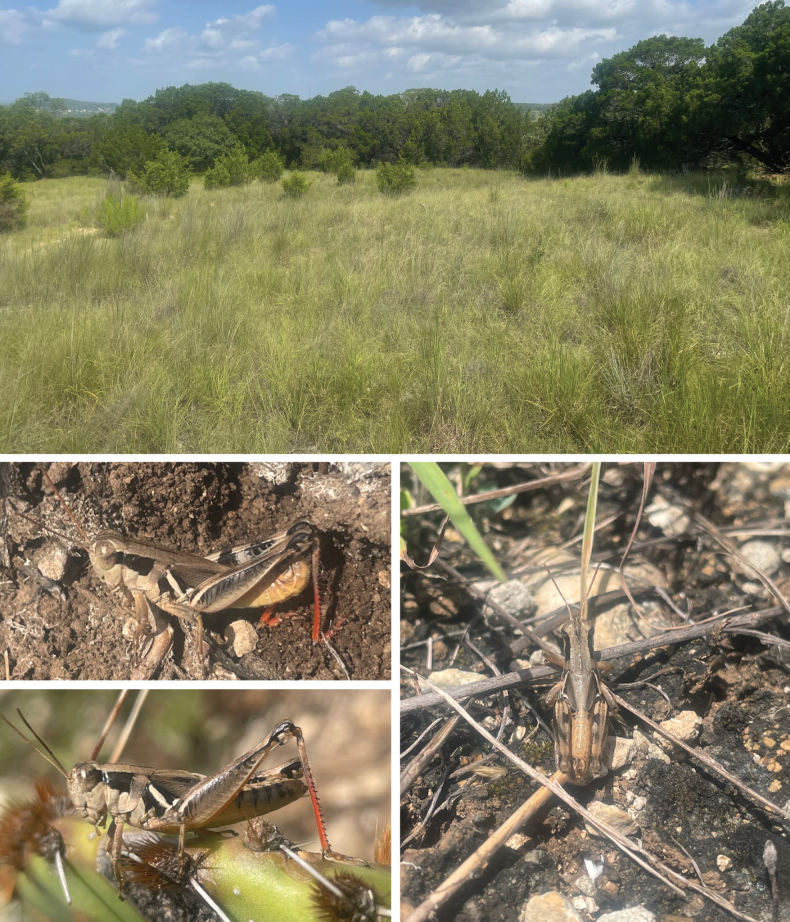
Habitat and live images of *Melanoplusbalcones* sp. nov. **A** Ashe juniper savanna, Travis Co., Texas **B** male **C** female **D** male.

Figs 2F , 3I , 4I , 9A–J , 14 , 19

##### Type material.

***Holotype***: 1♂, USA, TEXAS, Travis Co., 5.7 mi NW Lago Vista, 30.5041, -98.0678, 23 July 2021, J.G. Hill; Collected in live oak savanna and diverse grassland. Deposited in the Mississippi Entomological Museum.

##### Other specimens examined.

**Texas**: Travis Co., 5.7 mi NW Lago Vista, 30.5041, -98.0678, 23 July 2021, J.G. Hill (2♂).

##### Diagnosis.

Male cerci that are broadly falcate (Figs 2F, 9A, B), and the internal male genitalia with the aedeagal sheath that does not project to the distal edge dorsal valves. Dorsal valves are thin plates that have a caudal margin that is perpendicular to the sheath and then slopes downward apically to point, and are produced laterally to the ventral valves, giving the aedeagus a narrow or thin appearance in caudal or dorsal views (Figs 3I, 4I). The ventral valves are slightly shorter than the dorsal valves (Fig. 9F). Most similar to *M.kendalli and M.susdentatus*, but *M.balcones* is easily separated from *M.kendalli* by having an aedeagal sheath that does not reach the distal margin of the dorsal valves and the shape of dorsal valves (Fig. 9C–G), and from *M.susdentatus* by the longer, more arching dorsal aedeagus valves found in that species (Fig. 8C–G).

##### Male measurements.

(mm): (*n* = 3) Body length 19.4–20.6 (mean = 20); pronotum length 4.0–4.6 (mean = 4.3); tegmen length 4.0–4.6 (mean = 4.3); hind femur length 10.6–11.2 (mean = 10.9); cerci length 0.8–0.9 (mean = 0.9); basal width of cercus 0.6–0.7 (mean = 0.7); mid-cercal width 0.4–0.6 (mean = 0.5); cerci apex width 0.3 (mean = 0.3).

##### Habitat.

Live oak savanna or juniper oak savanna (Fig. 19A–C).

##### Distribution.

East central Balcones Escarpment in the vicinity of Travis County (Fig. 14).

##### Etymology.

Named after the Balcones Canyonlands and Escarpment where this species is endemic.

##### Suggested common name.

Balcones pouncer.

#### ﻿ Melanoplus corniculatus sp. nov.

Taxon classificationAnimaliaOrthopteraAcrididae

4DC1B741-DA50-5741-95D2-F5AAE3B91EE8

https://zoobank.org/E60D5951-BC1B-43D5-B1F4-17D17238B051

**Figure 20.**
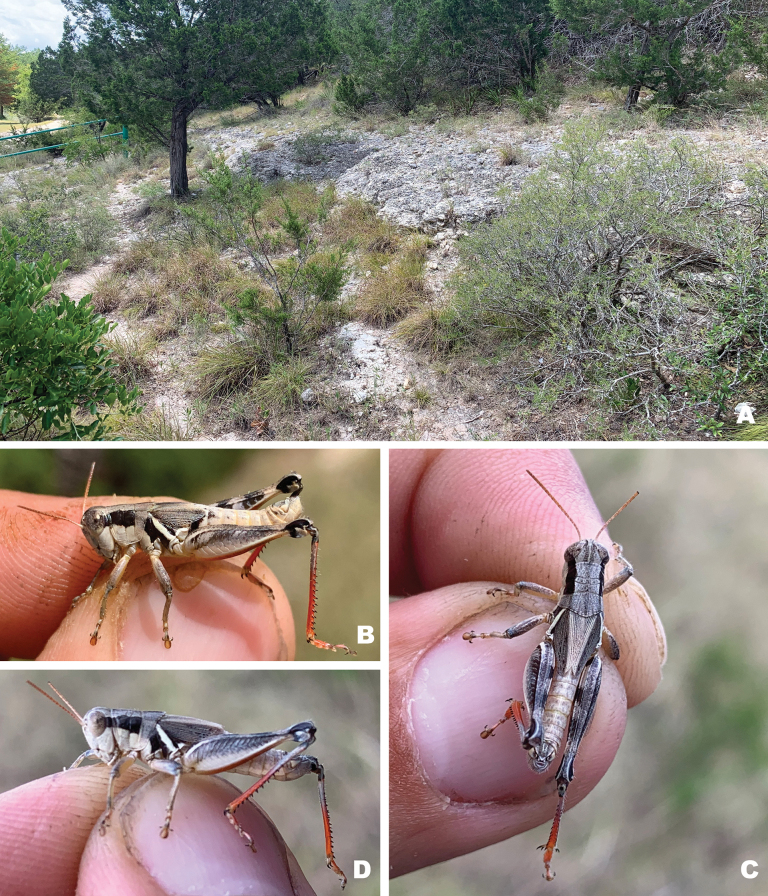
Habitat and live images of *Melanopluscorniculatus* sp. nov. **A** Ashe juniper savanna, Kerr Co., Texas **B** male **C** female **D** male.

Figs 2D , 3C , 4C , 10A–J , 14 , 20

##### Type material.

***Holotype***: 1♂, USA, TEXAS, Kerr Co., Kerrville, 30.0677223, -99.139329, 25 July 2018, J.G. Hill, Collected in oak juniper savanna. Deposited in the Mississippi Entomological Museum.

##### Other specimens examined.

**Texas**: Bandera Co., 4.5 mi N. Medina, 29.8502, -99.3030, 28 July 2020, J.G. Hill (2♂). Kerr Co., Kerrville, 30.0677223, -99.139329, 25 July 2018, J.G. Hill (1♂, 3F); 0.2 mi N Hunt, 30.0736, -99.3350, 27 July 2020, M.J. Thorn (6♂, 3♀); Same data, except B. S Dunaway (2♂); 3.7 mi E Hunt, 30.0576, -99.3965, 26 July 2020, M. J. Thorn (2♂, 1♀); Same data, except A. G. Hendon (1♂); 4.3 mi SW Hunt, 30.0310,-99.3945, 26 July 2020, M. J. Thorn (1♂); Same data, except A.G. Hendon (1♂).

##### Diagnosis.

Male cerci falcate with a steeply curving ventral edge (Figs 2D, 10A, B), internal male genitalia with the valves of the aedeagus projected caudally with dorsal valves that are trifid with the medial branch darker and longer than the distal two. (Figs 3C, 4C). Most similar to *M.walkeri*, but *M.corniculatus* is easily separated from that species by having a less steeply curving male cerci (Fig. 2D, E), aedeagal valves that are produced caudally instead of dorsally (Figs 10C–F, 11C–F), and the shape of the dorsal valves (Figs 10C–G, 11C–F).

##### Measurements.

**Male measurements.** (mm): (*n* = 14) Body length 19.0–22.3 (mean = 20.2); pronotum length 4.0–5.0 (mean = 4.4); tegmen length 4.8–6.0 (mean = 5.3); hind femur length 9.9–11.6 (mean = 11.0); cerci length 0.9–1.3 (mean = 1.0); basal width of cercus 0.6–0.9 (mean = 0.8); mid-cercal width 0.4–0.6 (mean = 0.5); cerci apex width 0.3–0.4 (mean = 0.3).

**Female measurements.** (mm): (*n* = 7) Body length 19.2–24.5 (mean = 22.1); pronotum length 4.5–5.3 (mean = 5.0); tegmen length 5.5–7.5 (mean = 6.2); hind femur length 11.1–13.3 (mean = 12.3); dorsal ovipositor valve length 1.3–1.7 (mean = 1.5); ventral ovipositor valve length 1.5–1.7 (mean = 1.5).

##### Habitat.

Ashe Juniper savanna (Fig. 20A).

##### Distribution.

Endemic to the Edwards Plateau of Texas in the vicinity of Kerr and Bandera Counties (Fig. 14).

##### Etymology.

From the Latin meaning horned (antlered) or horn shaped. In reference to the dorsal valves being shaped like antlers.

##### Suggested common name.

Antlered pouncer.

#### ﻿ Melanoplus walkeri sp. nov.

Taxon classificationAnimaliaOrthopteraAcrididae

4EA4A68E-F386-5D48-A9A9-1285382D4152

https://zoobank.org/3F32C8A6-106F-4D0E-8668-317787654E45

**Figure 21.**
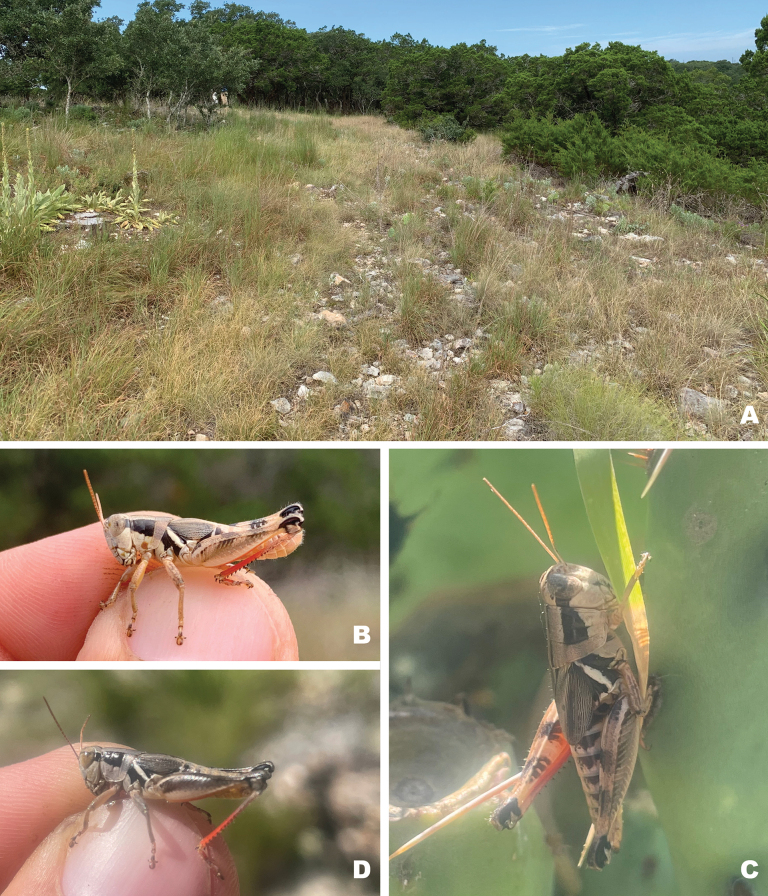
Habitat and live images of *Melanopluswalkeri* sp. nov. **A** Ashe juniper savanna, Kendall Co., Texas **B** male, **C** male **D** female.

Figs 2E , 3G , 4G , 11A–J , 14 , 21

##### Type material.

***Holotype***: 1♂, USA, TX, Kendall Co., 7 mi NE Sisterdale, 30.0073, -98.5515, 22 July 2019, J. G. Hill, MJT, BSD; Collected on Ashe juniper hill; 281,486. Deposited in the Mississippi Entomological Museum.

##### Other specimens examined.

**Texas**: Kendall Co., 7 mi NE Sisterdale, 30.0073, -98.5515, 22 July 2019, J. G. Hill, M.J. Thorn, B.S. Dunaway (3♂, 3♀).

##### Diagnosis.

Male cerci that are falcate with a very steeply curving ventral edge (Figs 2E, 11A, B), internal male genitalia with the valves of the aedeagus projected dorsally with trifid, thinly sclerotized dorsal valves. (Figs 3G, 4G). Most similar to *M.corniculatus*, but *M.walkeri* is easily separated from that species by having more steeply curving male cerci (Fig. 2D, E), aedeagal valves that are produced dorsally instead of caudally (Figs 10C–F, 11C–F), and the shape and level of sclerotization of the dorsal valves (Figs 10C–G, 11C–F).

##### Measurements.

**Male measurements.** (mm): (n = 4) Body length 21.4–22.1 (mean = 21.7); pronotum length 4.2–5.0 (mean = 4.7); tegmen length 4.5–5.7 (mean = 5.1); hind femur length 11.2–11.9 (mean = 11.6); cerci length 0.9 (mean = 0.9); basal width of cercus 0.8–0.9 (mean = 0.9); mid-cercal width 0.5 (mean = 0.5); cerci apex width 0.3 (mean = 0.3).

**Female measurements.** (mm): (*n* = 3) Body length 22–24.2 (mean = 22.8); pronotum length 5.5–5.7 (mean = 5.6); tegmen length 6.0 (mean = 6.0); hind femur length 13.3–13.5 (mean = 13.4); Dorsal ovipositor valve length 1.4–1.6 (mean = 1.5); ventral ovipositor valve length 1.3–1.5 (mean = 1.4).

##### Habitat.

Ashe juniper/live oak savanna.

##### Distribution.

Found in the central eastern edge of the Edwards Plateau along the Balcones Escarpment (Fig. 14).

##### Etymology.

Named in honor of Jerry Jeff Walker an iconic Texas musician whose most influential album was recorded near the type locality of this species in the Luckenbach. Walker’s songs such as Hill Country Rain, Leavin’ Texas, and Sangria Wine brought me and my field team joy while traveling between field sites and added to the amazing ambiance of the Edwards Plateau.

##### Suggested common name.

Walker’s pouncer.

#### ﻿ Melanoplus comanche sp. nov.

Taxon classificationAnimaliaOrthopteraAcrididae

B060BF7D-E94F-5650-AFF5-CC53F3F1BFD4

https://zoobank.org/EE160E4D-5699-4F7F-A671-36A5E1D2703F

Figs 2G , 3D , 4D , 12A–J , 14

##### Type material.

***Holotype***: 1♂, USA, TEXAS, Bandera Co., 4.5. mi NE Bandera, 29.7646, -99.0346, 25 August 2022, J.G. Hill; Collected in Ashe juniper savanna, MEM 501,225. Deposited in the Mississippi Entomological Museum.

##### Other specimens examined.

**Texas**, Bandera Co., 4.5 mi NE Bandera, 29.7646, -99.0346. 26 August 2022, J.G. Hill (4♂); 7 mi NE Bandera, 29.7312, -98.8711, 17 July 2019, J.G. Hill, M.J. Thorn, B.S. Dunaway (4♂, 4♀). Medina Co., 9 mi N Rio Medina, 29.5626, -98.8823, 17 July 2019, J.G. Hill, M.J. Thorn, B.S. Dunaway (4♂, 3♀).

##### Diagnosis.

Male cerci broadly falcate (Figs 2G, 12A, B), internal male genitalia where the valves of the aedeagus projected dorsally, appearing very broad in dorsal or caudal view, and with the dorsal valves forming semi-circular like tubes (Figs 3D, 4D). Most similar to *M.kendalli*, but *M.comanche* is easily separated from that species by having a much broader aedeagus in dorsal or caudal view and dorsal aedeagal valves that are form semi-circular tube as opposed to being flattened in *M.kendalli* (Figs 8C–F, 12C–F), and the shape and level of sclerotization of the dorsal valves (Figs 8C–G, 12C–F).

##### Measurements.

**Male measurements.** (mm): (*n* = 6) Body length 18.2–21.2 (mean = 19.5); pronotum length 4.1–4.9 (mean = 4.5); hind femur length 9.6–12.0 (mean = 10.8); cerci length 0.8–0.9 (mean = 0.9); basal width of cercus 0.5–0.6 (mean = 0.6); mid-cercal width 0.4 (mean = 0.4); cerci apex width 0.2–0.3 (mean = 0.3).

**Female measurements.** (mm): (*n* = 3) Body length 21.5–24.3 (mean = 22.9); pronotum length 4.5–5.7 (mean = 5.1) tegmen length 4.7–6.5 (mean = 5.7); hind femur length 11.5–13.7 (mean = 12.9); Dorsal ovipositor valve length1.4–1.5 (mean = 1.5); ventral ovipositor valve length 1.3–1.4 (mean = 1.4).

##### Habitat.

Oak savanna, Ashe juniper savanna and along a dry creek bed in an Ashe juniper savanna.

##### Distribution.

Southern Edwards plateau in the vicinity of Bandera and Medina Counties (Fig. 14).

##### Etymology.

Named in honor of the Comanche tribe of Native Americans who previously inhabited the area where this species occurs.

##### Suggested common name.

Comanche pouncer.

#### ﻿ Melanoplus tonkawa sp. nov.

Taxon classificationAnimaliaOrthopteraAcrididae

30AE8CB1-9623-5BC6-9460-DBEF53890E65

https://zoobank.org/BF2D0335-B602-4800-A831-037CDD619751

Figs 2G , 3H , 4H , 13A–J , 14

##### Type material.

***Holotype***: 1♂, USA, TX, 1.5 mi NNW Ellinger, 29.8511, -96.7173, 14 July 2020, J.G. Hill; collected in post oak savanna. Deposited in the Mississippi Entomological Museum.

***Paratypes*: Texas**: Fayette Co., 1.5 mi NNW Ellinger, 29.8511, -96.7173, 14 July 2020, J.G. Hill (2♂).

##### Diagnosis.

Male cerci broadly falcate (Figs 2G, 13A, B), internal male genitalia with the aedeagal sheath that does not project to the distal edge dorsal valves. Dorsal valves are thin plates that are arched along the caudal margin and are produced laterally to the ventral valves, giving the aedeagus a narrow or thin appearance in caudal or dorsal views (Figs 3H, 4H). The ventral valves are slightly shorter than the dorsal valves, are slightly arched posteriorly and have their distal ends bent medially (Fig. 13F). Most similar to *M.kendalli*, *M.balcones*, and *M.susdentatus*. *Melanoplustonkawa* is easily separated from *M.kendalli* by having an aedeagal sheath that does not reach the distal margin of the dorsal valves and the curved nature of the dorsal valves (Figs 7C–G, 13C–G), from *M.balcones* by the shorter, rounded valves found in that species (Figs 9C–F, 13C–F), and from *M.susdentatus* by the more rounded apices of the male cerci, less sclerotized and less broadly arching dorsal valves of the aedeagus (Figs 8C–G, 13C–G).

##### Male measurements.

(mm): (*n* = 2) Body length 20–21 (mean = 20.5); pronotum length 4.2–4.5 (mean = 4.4); hind femur length 11.2–11.4 (mean = 11.3); cerci length 1.0–1.2 (mean = 1.0); basal width of cercus 0.6 (mean = 0.6); mid-cercal width 0.4 (mean = 0.4); cerci apex width 0.3 (mean = 0.3).

##### Habitat.

Post oak/live oak savanna.

##### Distribution.

In the vicinity of Fayette County Texas on the western edge of the North American Coastal Plain (Fig. 14).

##### Etymology.

Named in honor of the Tonkawa tribe of Native Americans who previously inhabited the area where this species occurs.

##### Suggested common name.

Tonkawa pouncer.

## ﻿Discussion

The new species described here all come from known biodiversity hotspots, The Edwards Plateau and the North American Coastal Plain. The biological richness of the North American Coastal Plain has been well documented (Noss et al. 2014; Noss 2016; Hill 2018) and the discovery of a new grasshopper species (*M.tonkawa*) there is unsurprising.

The apparent center of diversity of the *M.discolor* group is on the Edwards Plateau and more specifically along the Balcones Escarpment, where five of the seven species occur, further demonstrating the high level of endemism on the Edwards Plateau. A phylogeny for the *discolor* group is not available at present. As such, the origins of the group remain uncertain. Many other brachypterous groups of North American melanoplines appear to have speciated due to glaciation and climatic changes during the Pleistocene that affected river flow, mountain ecosystems, or isolation of islands/sand ridges (Knowles 2001; Woller 2017; Huang et al. 2020). Toomey et al. (1993) describes the environment of the Edwards Plateau during the late Pleistocene (ca. 20–14,000 yr B.P.) as having much of the uplands covered in a deep reddish-clay soil and open mixed tall and short grass savanna. Drying conditions during the Holocene (10,500–2,500 yr B.P) resulted in diminished vegetation cover which caused the gradual degradation of soil mantles and a shift to short grasses and scrub plant communities (Toomey et al. 1993).

No clear biogeographic patterns are evident for the *discolor* group on the Balcones Escarpment, with some species being found on either sides of large rivers and other potential barriers. However, species in the *discolor* group are inhabitants of short grass communities, and I hypothesize that the drying conditions during earlier Pleistocene interglacial periods allowed the ancestral forms to disperse northward. The populations would have then been isolated during following glacial periods by the dissected landscape along the Balcones Escarpment and in some cases large river systems. The resulting plant community changes from the drying conditions during the late Pleistocene and Holocene would have allowed for the spread of the already differentiated populations, which may explain why some populations arose so close to each other without any apparent geographical barriers.

A complete phylogeny of the North American Melanoplinae is under way but is still several years away from completion. Population genetic data from each *discolor* group species are being included in that study. Hopefully, the results of that phylogenetic analysis will shed more insight into this interesting diversification event.

Despite being relatively secure in terms of conservation at present, *discolor* group species are facing increasing threats. King Ranch bluestem (Bothriochloaischaemumvar.songarica) is an invasive grass that is rapidly replacing native grasses in both disturbed and undisturbed shortgrass communities on the Edwards Plateau. In general, grasshopper abundance and diversity are low where this grass is abundant (pers. obs.). The historical abundance levels of Ashe juniper (*Juniperusashei*) have been contested for some time, but what is evident is that it does seem to be increasing in abundance in recent years, likely due to fire suppression (Gelbach 1988). Over time, the increasing Ashe juniper canopy cover shades out the short grasses, reducing grasshopper forage. More recently, human population levels along the Balcones Escarpment, especially around the city of Austin have grown immensely. Here former rangelands have been converted to suburban neighborhoods. Several times I have been able to find populations of species in the *discolor* group in the unmanaged sections of lawns in these neighborhoods where the original plant community was not overly disturbed during construction. These grasshoppers may be able to persist as long as the landowners maintain an open landscape and do not replace the native grasses and forbs with exotic species.

## Supplementary Material

XML Treatment for Melanoplus discolor

XML Treatment for Melanoplus nelsoni

XML Treatment for Melanoplus kendalli

XML Treatment for Melanoplus susdentatus

XML Treatment for Melanoplus balcones

XML Treatment for Melanoplus corniculatus

XML Treatment for Melanoplus walkeri

XML Treatment for Melanoplus comanche

XML Treatment for Melanoplus tonkawa

## ﻿Additional information

### Author ORCIDs

JoVonn G. Hill https://orcid.org/0000-0002-1892-7117

### Data availability

All of the data that support the findings of this study are available in the main text or Supplementary Information.
